# Cultural values predict national COVID-19 death rates

**DOI:** 10.1007/s43545-021-00080-2

**Published:** 2021-03-09

**Authors:** Damian J. Ruck, Joshua Borycz, R. Alexander Bentley

**Affiliations:** 1 Advai Ltd, 20-22 Wenlock Road, London, N1 7GU UK; 2 Sarah Shannon Stevenson Science and Engineering Library, Vanderbilt University, Nashville, TN 37203 USA; 3 Anthropology Department, University Tennessee, Knoxville, TN 37996 USA

**Keywords:** Computational social science, Development, Cultural evolution, Health policy

## Abstract

National responses to a pandemic require populations to comply through personal behaviors that occur in a cultural context. Here we show that aggregated cultural values of nations, derived from World Values Survey data, have been at least as important as top-down government actions in predicting the impact of COVID-19. At the population level, the cultural factor of cosmopolitanism, together with obesity, predict higher numbers of deaths in the first two months of COVID-19 on the scale of nations. At the state level, the complementary variables of government efficiency and public trust in institutions predict lower death numbers. The difference in effect between individual beliefs and behaviors, versus state-level actions, suggests that open cosmopolitan societies may face greater challenges in limiting a future pandemic or other event requiring a coordinated national response among the population. More generally, mass cultural values should be considered in crisis preparations.

**Supplementary Information:**

The online version contains supplementary material available at 10.1007/s43545-021-00080-2.

## Introduction

Combating the COVID-19 pandemic in nations around the world has depended on accurate information (e.g., Prather et al. 2020) guiding responses at different levels, including government response (Aksoy et al. 2020; Bedford et al. 2020; Chowell and Mizumoto 2020; Frey et al. 2020; Hale et al. 2020; Munster et al. 2020; Zhang and Qian 2020) as well as individual behaviors (Funk et al. 2010; Guiteras et al. 2015; Maharaj and Kleczkowski 2012; Zhang and Centola 2019). Since culture is the context for behavior (Gelfand et al. 2020; Muthukrishna 2020; Zhang and Centola 2019)—social scientists maintain that culture fundamentally determines behavior, values, beliefs, and even perceived reality in a society (Cronk 1999; Durkheim 1915; Henrich 2015)—effectiveness of intervention on COVID-19 by national governments ought to reflect cultural values among the people of those countries. As cultural values vary substantially across the world (Aksoy et al. 2020; Inglehart and Welzel 2005; Ruck et al. 2020a), there is a motivation to ascertain whether cultural values predict national COVID-19 rates.

Recent studies have used socioeconomic and public health variables to explain COVID-19 variation within the United States (Desmet and Wacziarg 2020) and also globally (de Oliveira et al. 2020). In the context of the literature on the effect of socioeconomic factors on COVID-19 (Aksoy et al. 2020; Bedford et al. 2020; Chowell and Mizumoto 2020; Frey et al. 2020; Funk et al. 2010; Guiteras et al. 2015; Hale et al. 2020; Maharaj and Kleczkowski 2012; Munster et al. 2020; Zhang and Centola 2019; Zhang and Qian 2020), we use unique measures of national-scale cultural values, derived from multivariate study of decades of World Values Survey results (Ruck et al. 2018, 2020a, b). We examine these cultural effects in concert with known risks such as obesity and advanced age, together with variables describing government efficiency and public trust in institutions.

Here, we estimate the observable effects of these factors in different countries on national COVID-19 fatality rates. We assume deaths to be a delayed proxy for true number of cases (Baud et al. 2020; Birrell et al. 2020; Marchant et al. 2020), as fatality data are generally more reliable than case data. We use the COVID-19 fatality data from the European Centre for Disease Prevention and Control (Rosler et al. 2020). Although reported death counts have some uncertainty in terms of different government reporting structures (Economist 2020), alternatives such as data on excess deaths have not only reporting uncertainties but are also incomplete and scattered on the global scale (e.g., the Human Mortality database is limited to 33, mostly Western, countries with missing data).

The national-scale cultural factors are derived from recent work, applying a two-stage factor analysis to World and European Values Survey data (WVS 2020, EVS 2011) in 109 countries (Ruck et al. 2020a, b). The cultural factors we consider here are secular-rationality (RAT), cosmopolitanism (COS) and institutional trust (INST). We expect institutional trust (INST) within a population, which is likely to help government efforts to mitigate a pandemic (Aksoy et al. 2020), may exhibit effects in concert with the government efficiency variable. Secular-rationality (RAT) is correlated with secularism, political engagement (and critique), and respect for individual rights, but also low prosociality (Ruck et al. 2020a). We hypothesize that RAT would predict higher epidemic spread (and hence more deaths) among people who are more individualistic and perhaps more heterogeneous in how they follow guidelines. We also predict that the cultural factor of Cosmopolitanism (COS) would, all else being equal, predict more coronavirus deaths via the effect of personal intermixing. Populations with high Cosmopolitanism (COS) are more open to have neighbors that are foreign or of a different ethnicity (Ruck et al. 2018, 2020a).

We entered these cultural factors into a matrix of country-scale covariates, ***X***, among the 88 counties for which we also have data for government efficiency from an established index (WEF 2018; Mohamadi et al. 2017), as well as the logarithm of GDP per capita, population size, per cent urban population, obesity and per cent of population aged 65 and over (see Materials and Methods). We account for the demographic factors of population size and fraction aged over 65 by including these as covariates in the matrix. We also include national rates of obesity, which increases risk of fatality (Hamer et al. 2020) and fraction urban population, as clustering facilitates spread of COVID-19 (Yusef et al. 2020). We also include Gross Domestic Product (GDP), the effects of which will probably be commingled with obesity and longevity, which correlate with economic development.

We first explore the variance structure in the covariate matrix, ***X***, by principal component analysis. We look at the component loadings and see how the first two principal components correlate with per-capita COVID-19 deaths. Subsequently, we use multivariate regression to explain how the individual covariates predict the residual variance in COVID deaths in the different countries $\vec{N}_{d}$:

1 $${\text{log(}}\vec{N}_{d} ) = \vec{\beta }{\varvec{X}} + \epsilon,$$

where the errors *ϵ* follow a negative binomial distribution and have a variance for a given mean, *µ*, of *µ*(1 + *µ*/*r*), where *r* is a dispersion parameter. The numbers of COVID deaths, $\vec{N}_{d}$, are daily-updated national counts (Rosler et al. 2020) through the first two months of the outbreak in each country.

Since COVID-19 deaths are count data that are highly dispersed, we model negative-binomial distributed errors, using the “glmmADMB” package in R (Bolker et al. 2012), rather than a more restrictive Poisson distribution. The regression determines the vector of country-specific coefficients, $\vec{\beta }$, applied to the covariate matrix ***X*** (Eq. 1). While we consider the covariates as constants for the duration of the pandemic, the number of COVID-19 deaths grew exponentially so we fit regressions at one-day intervals from 5 to 55 days after the day when the first death occurred in each country. To ensure the results of these regressions are not sensitive to the 87-country sample, we find broadly the same results using an ensemble of bootstrapped samples (see Supplementary Materials).

## Results and discussion

**Fig. 1:**
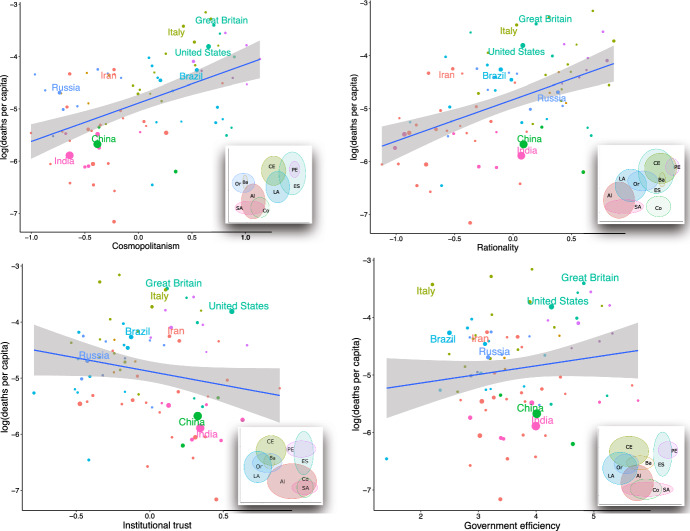
COVID-19 spread versus cultural factors. Main plots show individual countries and LOESS correlation. Insets show 1 S.D. confidence ellipses by cultural region (Inglehart and Baker 2000; Ruck et al. 2020b): *AI* African-Islamic, *Ba* Baltic, *CE* Catholic Europe, *Co* Confucian, *ES* English Speaking, *LA* Latin America, *PE* Protestant Europe, *Or* Orthodox, *SA* South Asia

We start by visualizing simple correlations that will subsequently be unpacked through mutivariate analysis. As Fig. 1 shows, the initial spread of COVID-19 was positively correlated with cosmopolitanism, COS (Adjusted *r*^2^ = 0.229, *p* < 0.0001) and rational-secularism, RAT (Adj. *r*^2^ = 0.217, *p* < 0.0001). Deaths correlate negatively with institutional confidence, INST (Adj. *r*^2^ = 0.029, p = 0.066). There is little correlation, however, with government efficiency, but it is positive (Adj. *r*^2^ = 0.011, *p* = 0.168).

The first two principal components (PCs) from the principal component analysis, which together explain 67% of the variance (Table 1), appear to capture two main sources of variation: people and institutions. The first component, PC1, which accounts for 46% of the variance, is positively loaded on all covariates except INST, and not strongly loaded on government efficiency (Table 1, Figs. S1 and S2). The second component, PC2, which reflects 21% of the variance, is primarily loaded on institutional confidence (INST) and government efficiency (Gov. eff.). To ensure that our choice of government variable did not determine the outcome, we tested the effects on the PCA using additional, similar variables (see Supplement).

**Table 1:** Variable loadings of the first four principal components (PC), with their % variance explained (in parentheses)

	PC1 (46.1%)	PC2 (21.1%)	PC3 (13.0%)	PC4 (7.4%)
RAT	0.746	− 0.156	0.538	0.098
COS	0.701	0.115	0.048	− 0.693
INST	− 0.046	0.902	0.108	0.077
Gov. eff	0.467	0.751	0.043	0.152
Urban	0.781	0.067	− 0.480	− 0.014
GDP	0.898	0.176	− 0.142	0.141
Age 65+	0.728	− 0.319	0.514	0.133
Obesity	0.691	− 0.363	− 0.465	0.192

GDP is logarithm of GDP per capita; Age 65+ is fraction of population aged 65 and older. Details of all variables are in Materials and Methods

**Fig. 2:**
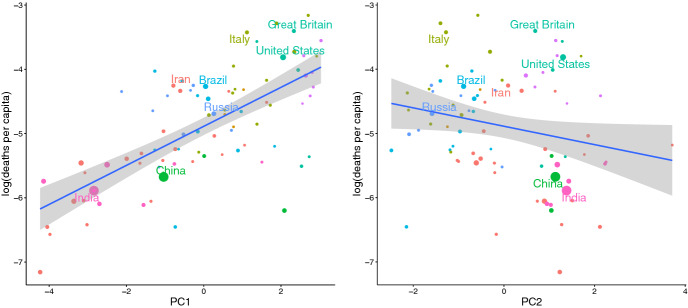
COVID-19 deaths vs (left) PC 1 and vs. (right) PC 2

Venturing to interpret the PCA, it appears that PC1 is a “people” component—cultural values, age and obesity—whereas PC2 captures the effectiveness of institutions, requiring effective governments and people with confidence in their institutions. The left side of Fig. 2 shows that COVID-19 deaths per capita correlate positively with PC1 (Adj. *r*^2^ = 0.469, *p* < 0.0001) and negatively with PC2 (Adj. *r*^2^ = 0.037, *p* = 0.045). A negative, but weaker, correlation with PC2 (Fig. 2, right) indicates that effective institutions predict reduced COVID-19 deaths per capita. As PC1 is also loaded on urbanism and GDP (Table 1), which themselves are underpinned by secular-rational and cosmopolitan cultural values (Ruck et al. 2018, 2020a), the comingling of factors motivate our regression analysis.

Next, we explore these hypotheses raised by the PCA with multivariate regression, which helps disentangle the joint effects of cultural factors, government efficiency, socioeconomic development and individual-level risks of age and obesity. In the regression results shown in Table 2, we have combined the institutional confidence and government efficiency variables as an interaction term, labelled as INST.Gov, as these combined variables are the essence of PC2, and also because we would expect governments to be more effective with public trust in their institutions. While Table 2 shows that INST.Gov has significant effect, the Supplementary materials show that institutional confidence and government efficiency have limited effect independently (Table S1), even with the independent effects included (Table S2).

**Table 2:** Results of negative binomial regression

Covariate (in matrix *X*)	Day 10	Day 50
(Intercept)	− 9.73 (2.45)***	− 18.38 (3.02)***
RAT	− 0.37 (0.30)	0.46 (0.37)
COS	0.08 (0.22)	1.01 (0.26)***
Urban	0.00 (0.01)	− 0.02 (0.01)
log(GDP)	1.17 (0.61)^†^	1.06 (0.78)
log (pop)	0.92 (0.18)***	2.44 (0.20)***
INST.Gov	− 0.04 (0.02)*	− 0.04 (0.02)^†^
SARS	− 0.05 (0.28)	0.19 (0.34)
Obesity	0.01 (0.01)	0.07 (0.01)***
Age 65+	0.05 (0.03)	0.03 (0.04)
Dispersion: parameter	1.56	1.02
AIC	660	1222
Observations	88	88

Predictors of COVID-19 deaths, 10 and 50 days after outbreak. In parentheses are heteroskedastic standard errors (for negative-binomial-distributed errors)

Heteroskedastic adjusted significance: ***p < 0.001, *p < 0.05, ^†^*p* < 0.10

The regression (Table 2) shows increased effect of several covariates on COVID-19 deaths per capita between Day 10 and Day 50, likely because so many fatalities occur weeks after infection. The *z*-scores of these effects between 5 and 55 days after the outbreak (Figure S3) increased over the first two months of the outbreak. By Day 50, the significant factors are more clear in the regression (Table 2). Not surprisingly, obesity and population size predict more deaths per capita. The Cosmopolitan (COS) cultural factor also predicts increased deaths. The combined variable, INST.Gov, predicts fewer deaths per capita (Table 2), which is broadly consistent with the negative correlation between PC2 and deaths (Fig. 2, right).

The regression results reveal similar patterns as the PCA. At the level of national populations, the cultural factor of cosmopolitanism, together with obesity, predict higher numbers of per-capita deaths in the first two months of COVID-19. At the government level, the complementary variables of government efficiency and public trust in institutions predict lower death numbers, but with weaker effect.

The measurable effect of the COS factor is larger after 50 days than after 10 days (Table 2), potentially because deaths increased by order(s) of magnitude during that time in many countries. We focus on Day 50 in Table 2, when the factor COS exhibits a larger effect than RAT (secular-rationalism). Strictly speaking, COS is a predictive factor in COVID deaths, not necessarily the ultimate cause. COS probably captures a cultural openness to human interaction, which facilitates COVID spread. Given the importance of government action (Hale et al. 2020)—in imposing travel restrictions, lockdowns and/or suspensions of educational, commercial and religious activities (Flaxman et al. 2020; Hsiang et al. 2020)—a reasonable counter-argument is that COS merely predicts the ability of national governments to control the pandemic. The problem with this is that authoritarian governments are better at imposing restrictions, and authoritarianism tends to correlate negatively with COS (Ruck et al. 2020a). Another counter-argument might be that nations with lower COS are less prone to accurately/honestly report their COVID statistics, and hence higher COS predicts higher *reported* COVID-19 deaths. This is possible and should be the subject of more granular, qualitative research.

Among demographic controls, population size and obesity predicted more deaths. By Day 50, obesity had the largest effect on COVID-19 deaths (Table 2). This was expected, as obesity increases the risk of fatality from COVID-19 (Hamer et al. 2020). Early in the outbreak (Day 10), higher GDP per capita predicts higher COVID-19 death rates (Table 2). While GDP correlates with obesity and life expectancy, this additional effect of GDP on COVID-19 may reflect economic incentives of wealthier populations to resist shutdown measures.

More broadly, our results support the case that open and tolerant societies, which tend to be democratic (Ruck et al. 2020a), may make it harder for governments to effectively mitigate the effects of a pandemic such as COVID-19. It might be related to the degree to which “survival” is a prominent factor in cultural values, in the sense that survival values prioritize economic and physical security over self-expression and quality of life, which tends to be more common in autocratic countries (Inglehart and Baker 2000). There is also a secondary effect of institutional confidence predicting lower deaths. All in all, this suggests that countries with high cosmopolitanism and low institutional confidence are in particular danger, such as many Latin American countries (Ruck et al. 2020a).

Finally, a larger question is the resilience of cultures and democracies to unprecedented challenges and events (Muthukrishna 2020). While trust in institutions predicted fewer COVID-19 deaths and ought to facilitate government action, this value has been declining for decades in many Western countries (Ruck et al. 2018). Cultural values of cosmopolitanism, which predict the economic prosperity and democracy of nations in the long term (Inglehart and Welzel 2005; Ruck et al. 2018, 2020a, b) may, in short-term crisis events, hinder a strategic, coordinated national response. Hence, while a multi-decade trend towards greater openness towards minorities around the world (Ruck et al. 2020a) is normally a good thing, governments should consider the role of cultural values in preparing for the next pandemic.

## Materials and methods

### Data on COVID-19 fatalities

COVID-19 deaths: data from the European Centre for Disease Prevention and Control (Rosler et al. 2020) were obtained from ourworldindata.com. Government efficiency index is taken from the World Economic Forum's 2018 Global Competitiveness (WEF 2018); it is a composite measure that quantifies: (1) efficient public spending, (2) weak burdens on private companies, (3) efficient judiciary, (4) responsive to private sector and (5) transparent policy changes. GDP per capita is measured at purchasing power parity (constant 2011 international dollars) for most recent year available for each country (World Bank 2018). Government response index is a composite variable of comprising information on 17 policies thought to help mitigate COVID-19 spread (Hale et al. 2020), including containment (school closures, mobility restrictions etc.), economic policies (e.g., direct payments) and heath policies (e.g., testing regimes, extra healthcare spending).

Methods for counting COVID-19 deaths vary between countries (e.g. including deaths at home as well as hospitals, likelihood of less effective counting in low income countries) as there is no internationally accepted standard. Cultural and political variables, which are inherent to our regressions, may also affect how COVID-19 deaths are counted. We nevertheless believe the effect of reporting differences to be small in our results. First, a plot of cumulative data from 25 Nov 2020, from 167 countries (with at least 1,000 reported cases) follow a linear slope (i.e., case fatality ratio, CFR) of 2.0% (*r*^2^ = 0.89). Notably, cumulative figures from the four income categories for nations (low, lower middle, upper middle, high) fall on this same line. Hence, if deaths at lower income levels were significantly under-reported, cases would have to be under-reported by the same percentage. The CFR across these categories ranges from 1.56% in low income countries to 3.09% in upper middle-income countries, suggesting some under-reporting of deaths in lower income countries. In log-transformed numbers, however, this is only a 15% difference, very slight on a log–log plot of cases versus deaths.

Furthermore, the outlier nations seem to reflect actual pandemic situation rather than reporting irregularities. The lowest CFRs are Singapore (0.048%), Curacao (0.16%), Qatar (0.17%), Botswana (0.31%), UAE (0.35%), and Maldives (0.36%), each of which appears to have been genuinely strict in controlling COVID-19, such as requiring visitors to show a negative result from a certified COVID-19 PCR-test. The highest CFRs are in Yemen (29%), Mexico (9.7%), Sudan (7.4%), Ecuador (7.1%) and Bolivia (6.2%), all of which are countries with genuinely, tragically high COVID-19 death rates as opposed to outstanding administrative protocols for reporting them. In summary, while there is uncertainty in the COVID-19 death numbers, we do not believe these uncertainties are systematic or large enough to explain our regression results.

### Control variables

Control variables were collated by ourworldindata.com (Rosler et al. 2020). These variables included percentage aged over 65 years from the World Bank's World Development Indicators, population sizes of nations in 2010 are from the United Nations Department of Economic and Social Affairs. Percentage urban population for nations in 2017 come from the World Bank's development indicators (Ritchie and Roser 2018). Obesity is measured as the percentage of the population aged over 18 that have a BMI greater than 30, using data from the World Health Organization (https://ourworldindata.org/obesity). We measured exposure to SARS during the 2002–2004 outbreak as a dummy variable, where a country is assigned a one if they had at least one case and a zero otherwise (WHO 2020). The countries effected by SARS are: China Hong Kong, Taiwan, Canada, Singapore, Vietnam', United States, Philippines, Thailand, Germany, France, Australia, Malaysia, Sweden, Great Britain, Italy, India, South Korea, Indonesia, South Africa, Kuwait, Ireland, New Zealand, Romania, Russia, Spain and Switzerland.

Cultural factors, including secular-humanism (RAT), openness to minorities (COS) and trust in institutions (INST), were derived from multivariate statistics and the World and European Values surveys (WEVS) data from 109 nations (EVS 2011; Inglehart and Welzel 2005; Ruck et al. 2018, 2020a, b; WVS 2020). The WEVS data are derived from the same 64 questions in the five waves of these surveys at 5-year intervals since 1990, administered to 476,583 participants from 109 different nations. These data were compressed into multivariate factors in two steps. The first used Exploratory Factor Analysis (EFA) to identify nine cultural factors underlying the WEVS data. From the EFA step, we summarized the common variance in the WEVS data and thereby remove the portion of the total variance that is likely to be measurement error or other forms of statistical noise. We then used the EFA factor loadings as weights for Principal Component Analysis (PCA), as the orthogonality of the principal components is advantageous for our subsequent regression modelling.

Here we have used the first three of these cultural components, labelled: Trust in Institutions, INST, Cosmopolitanism, COS, and Secular-Humanism, RAT (Ruck et al. 2020b). These components were interpreted based on the correlated cultural factors from the raw survey questions. Trust in Institutions, INST, was correlated with cultural factors such as confidence in institutions (*r* = 0.58) and interest in politics (*r* = 0.86). Individuals with high trust in institutions report high confidence in institutions like the media, the army and government and also have an active interest in politics. Secular-Humanism, RAT, is correlated with secularism (*r* = 0.76), political engagement (*r* = 0.62), respect for individual rights (*r* = 0.59) and low prosociality (*r* =  − 0.45) (Ruck et al. 2018). High RAT reflects survey respondents who reported, for example, that religion is not important in their lives, that they are likely to attend protests or sign petitions, they only pay taxes when coerced and believe that homosexuality and divorce are justifiable (Inglehart and Welzel 2005; Ruck et al. 2018). Cosmopolitanism, COS, is correlated with the exploratory cultural factors for ‘openness to out-groups’ (*r* = 0.78), ‘openness to norm violators’ (*r* = 0.78) and ‘subjective well-being’ (*r* = 0.43). High COS implies willingness to have neighbours that are immigrants, from another race, homosexual or from other stigmatized groups; as well as self-reporting happiness and life satisfaction (Ruck et al. 2018, 2020b).

### Principal component analysis

For principal component analysis (PCA), we use the ‘Factominer’ and ‘Factoextra’ packages in R to compute the contributions (Table S1) and loadings (Table S2) of the principal components. The PCA included all variables for 83 nations excluding Kosovo, Serbia and Montenegro, North Ireland, and Taiwan, which lacked urbanization data.

## Supplementary Information

Below is the link to the electronic supplementary material.

Electronic supplementary material 1 (PDF 465 kb)

## Data Availability

Original cultural factor data (Ruck et al. 2020a) generated for this research work have been archived within the Zenodo repository: https://doi.org/10.5281/zenodo.3559789. Additional covariate data analysed during the current study are available in these repositories: World Bank repository: https://data.worldbank.org/data-catalog/world-development-indicators. Our World in Data repositories for coronavirus statitstics: https://ourworldindata.org/coronavirus. Our World in Data repositories for urbanization: https://ourworldindata.org/urbanization. World Economic Forum Government Efficiency and Global Competitiveness Indices: http://reports.weforum.org/global-competitiveness-index-2017-2018.
