# Supplementary material for: Cultural values predict national COVID-19 death rates
Source: SN Soc Sci. 2021 Mar 9;1(3):74. doi: 10.1007/s43545-021-00080-2 (PMC7939727; doi:10.1007/s43545-021-00080-2)
Supplement: Supplementary file 1 — Electronic supplementary material 1 (PDF 465 kb) [file 43545_2021_80_MOESM1_ESM.pdf]

# Supplementary Materials: "Cultural values predict national COVID-19 death rates"

Damian J. Ruck,[1] Joshua Borycz[2], R. Alexander Bentley,[1*]

[1]Anthropology Dept., University Tennessee, Knoxville, TN, 37996 USA
[2]Sarah Shannon Stevenson Science and Engineering Library,
Vanderbilt University, Nashville, TN 37203 USA
[*]To whom correspondence should be addressed; E-mail: rabentley@utk.edu.

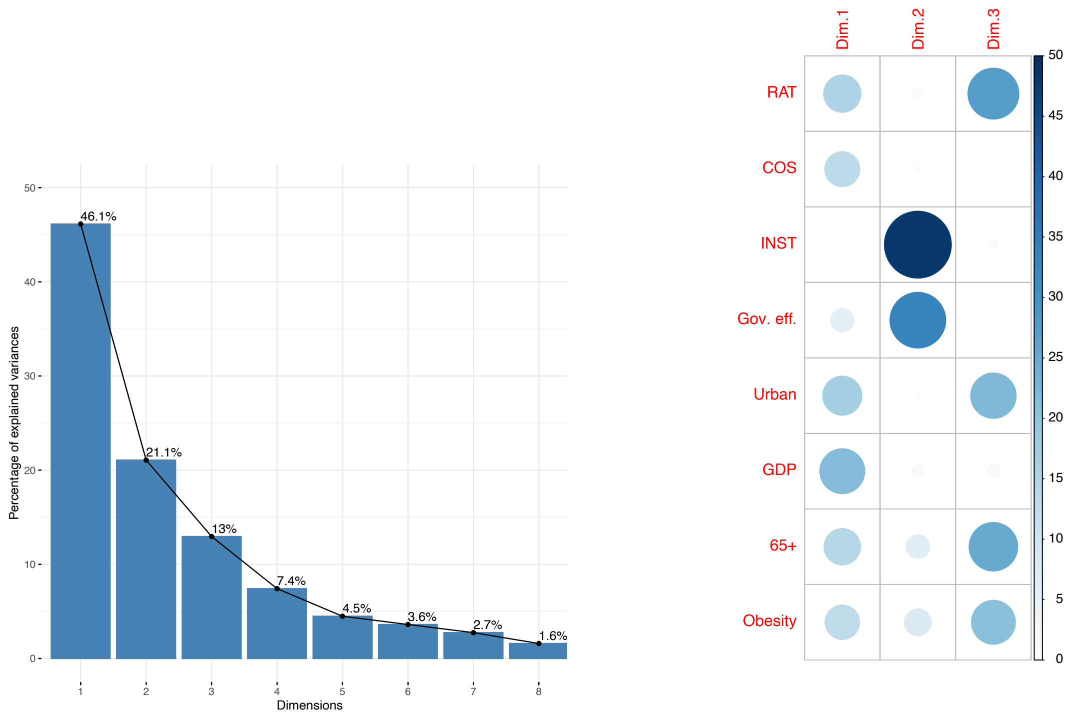


Figure S1: Left: Scree plot for the PCA analysis discussed in the text. Right: Bubble grid showing the relative loadings on the first three principal components.

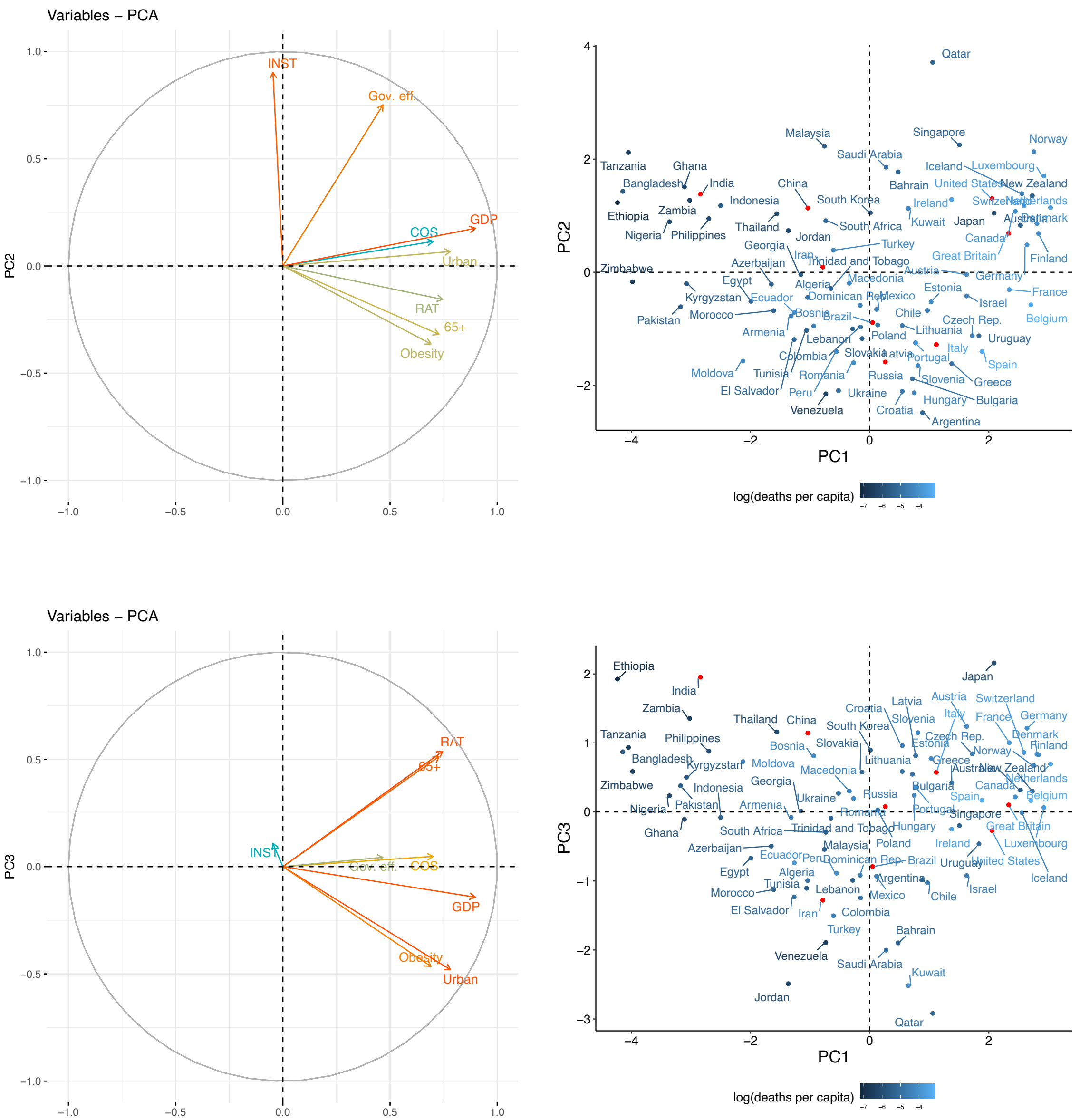


Figure S2: Principal components, PC1 vs. PC 2 on the top row and PC 1 vs. PC3 at bottom row. Plots on the left show how variables (see Table 1 for abbreviations) align with the PCs. Plots on right show PC scores for individual nations. Nations highlighted by red dots: Brazil, China, Great Britain, India, Italy, Iran, and U.S.

## Multi-variate regressions, no interaction terms

Table S1: Results of negative binomial regression. Predictors of COVID-19 deaths, 10 and 50 days after outbreak. In parentheses are heteroskedastic standard errors (for negative-binomial-distributed errors). Heteroskedastic adjusted significance: $^{***}p < 0.001$, $^{**}p < 0.01$, $^{*}p < 0.05$, $^{\dagger}p < 0.10$

| Covariate (in matrix $X$) | Day 10 | Day 50 |
|---|---|---|
| (Intercept) | $-9.69$ $(2.55)^{***}$ | $-18.21$ $(3.15)^{***}$ |
| RAT | $-0.39$ $(0.31)$ | $0.43$ $(0.37)$ |
| COS | $0.08$ $(0.22)$ | $0.99$ $(0.26)^{***}$ |
| INST | $-0.40$ $(0.39)$ | $-0.36$ $(0.46)$ |
| Urban | $0.00$ $(0.01)$ | $-0.02$ $(0.01)$ |
| log(GDP) | $1.07$ $(0.61)^{\dagger}$ | $0.93$ $(0.78)$ |
| log(pop) | $0.94$ $(0.19)^{***}$ | $2.46$ $(0.21)^{***}$ |
| Age 65+ | $0.05$ $(0.03)^{\dagger}$ | $0.04$ $(0.04)$ |
| Gov. Eff. | $-0.15$ $(0.16)$ | $-0.20$ $(0.19)$ |
| SARS | $-0.07$ $(0.29)$ | $0.18$ $(0.34)$ |
| Obesity | $0.01$ $(0.01)$ | $0.07$ $(0.01)^{***}$ |
| Dispersion: parameter | 1.54 | 1.01 |
| AIC | 663.19 | 1224.64 |
| Observations | 88 | 88 |

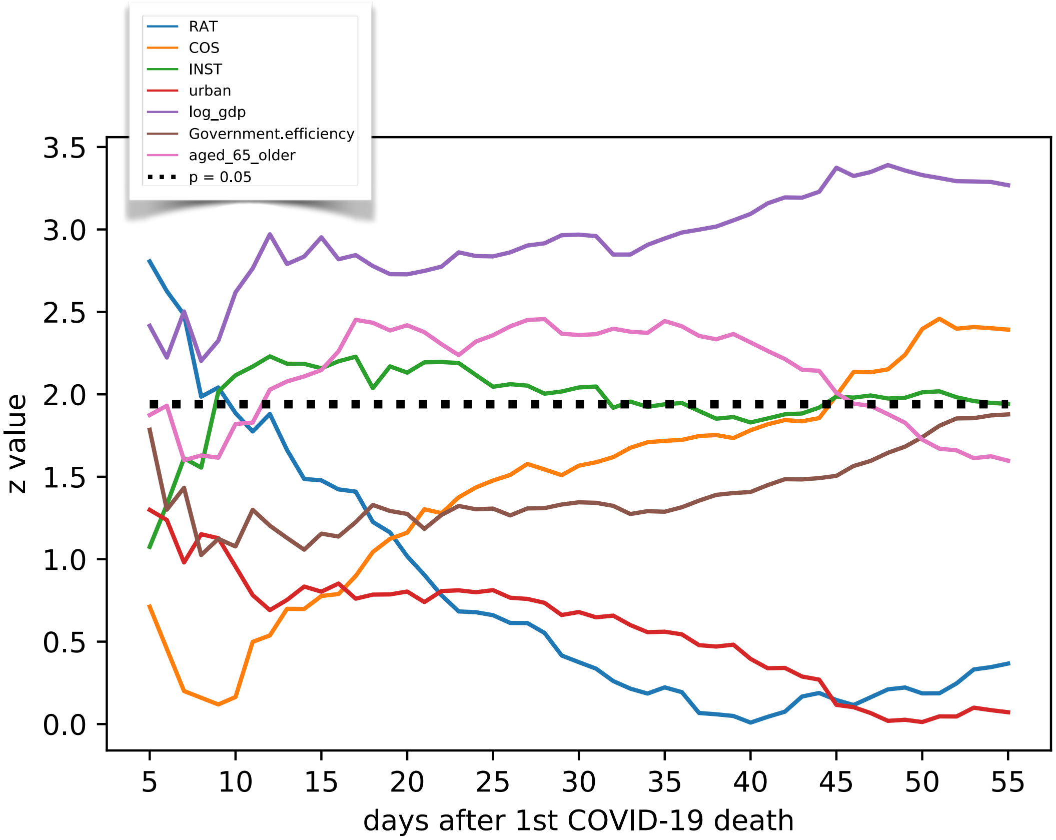


Figure S3: Significance of covariate effects on number of cases as the COVID-19 outbreak progresses (without interaction term); where z value = effect size/standard error. Dotted line indicates the z score corresponding with a p value of 0.05 in a two sided test.

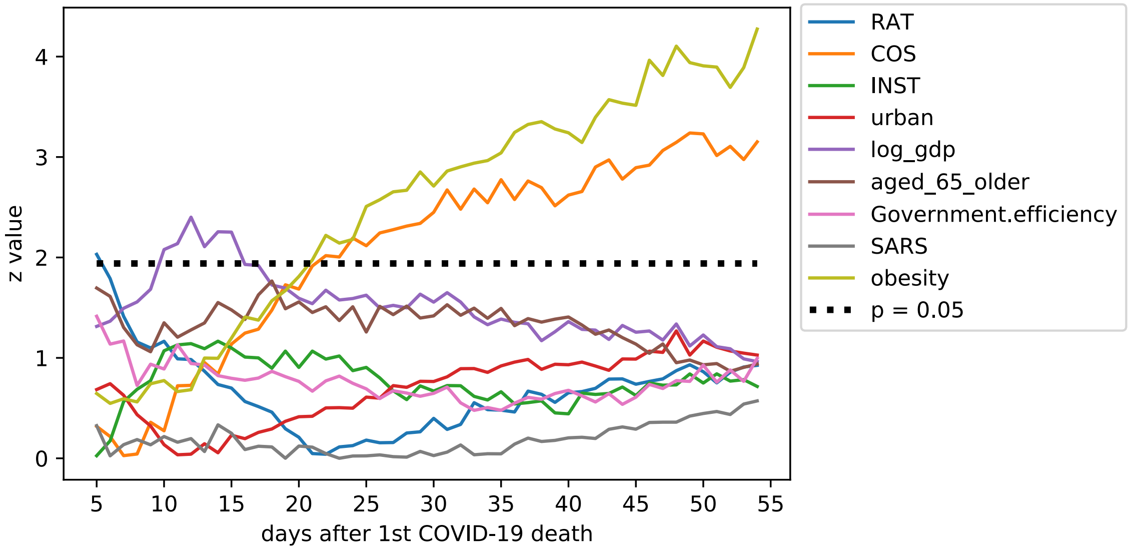


Figure S4: Significance of covariate effects using an ensemble of bootstrapped samples for parameter estimates (without interaction term). The z-score is effect size divided by standard error. Dotted line indicates the z-score corresponding with a two-sided $p$-value of 0.05.

## Multi-variate regressions with interaction terms

We ran multi-variate regressions with interaction between Government Efficiency (Gov. Eff.) and institutional Confidence (INST). Including an interaction term in the multivariate regression (Supplementary Table S2) caused the independent effects of INST and government efficiency two switch to positive, likely because they are highly co-mingled.

Table S2: Results of negative binomial regression. Predictors of COVID-19 deaths, 10 and 50 days after outbreak. In parentheses are heteroskedastic standard errors (for negative-binomial-distributed errors). Heteroskedastic adjusted significance: $^{***}p < 0.001$, $^{**}p < 0.01$, $^{*}p < 0.05$, $^{\dagger}p < 0.10$

| Covariate (in matrix $X$) | Day 10 | Day 50 |
|---|---|---|
| (Intercept) | $-14.64$ (3.60)$^{***}$ | $-24.08$ (4.23)$^{***}$ |
| RAT | $-0.40$ (0.30) | 0.52 (0.36) |
| COS | 0.12 (0.22) | 1.04 (0.26)$^{***}$ |
| INST | 0.82 (0.47)$^{\dagger}$ | 1.04 (0.54)$^{\dagger}$ |
| Urban | $-0.00$ (0.01) | $-0.02$ (0.01)$^{\dagger}$ |
| log(GDP) | 1.38 (0.62)$^{*}$ | 1.19 (0.78) |
| log(pop) | 0.91 (0.19)$^{***}$ | 2.43 (0.21)$^{***}$ |
| Age 65+ | 0.03 (0.03) | 0.01 (0.04) |
| Gov. Eff. | 1.21 (0.63)$^{\dagger}$ | 1.54 (0.77)$^{*}$ |
| SARS | $-0.06$ (0.28) | 0.17 (0.34) |
| Obesity | 0.02 (0.01) | 0.07 (0.01)$^{***}$ |
| INST.Gov | $-0.27$ (0.12)$^{*}$ | $-0.33$ (0.15)$^{*}$ |
| Dispersion: parameter | 1.63 | 1.06 |
| AIC | 660.84 | 1221.93 |
| Observations | 88 | 88 |

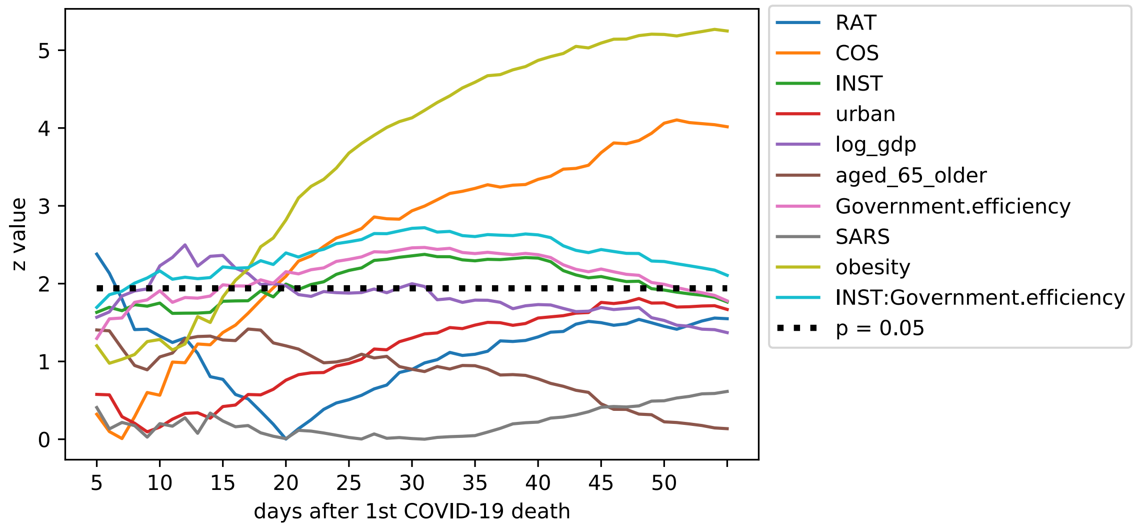


Figure S5: Significance of covariate effects on number of cases as the COVID-19 outbreak progresses. The z-score is effect size divided by standard error. Dotted line indicates the z-score corresponding with a two-sided $p$-value of 0.05.

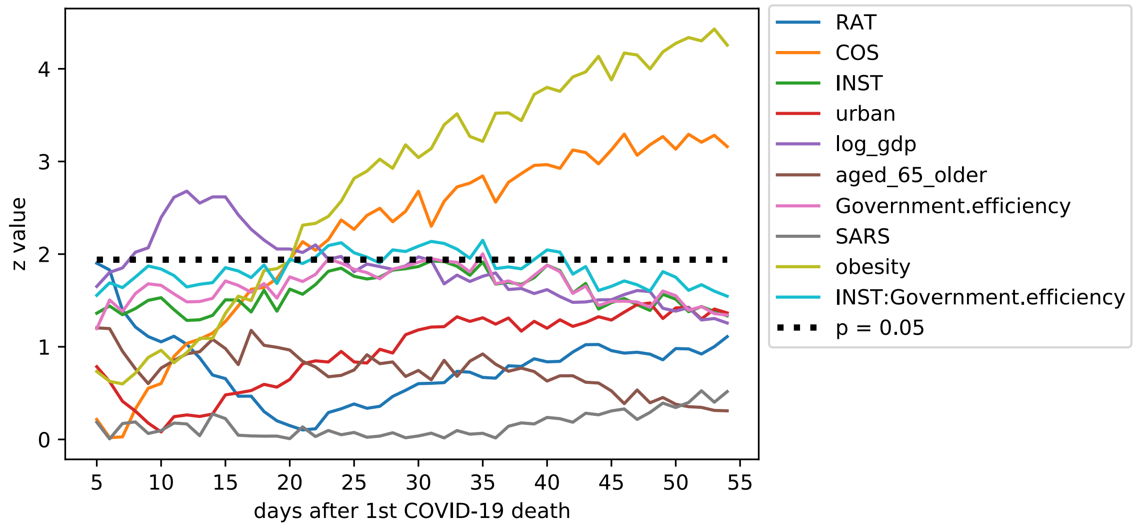


Figure S6: Significance of covariate effects using an ensemble of bootstrapped samples for parameter estimates. Dotted line indicates the z-score corresponding with a two-sided $p$-value of 0.05.
